# Electricity

**Published:** 1890-03-15

**Authors:** 


### THERAPEUTICS.

### ELECTRICITY.

Dr. George Herschell has some remarks on the abuse of electricity in the *Hospital Gazette*. Ignorant and designing charlatans have pretended to obtain the most absurd and extravagant results from its employment, and by so doing have brought undeserved neglect upon a remedy which has been proved to be of the greatest value in suitable cases. There is nothing magical in electricity that it will cure disease however it is applied, as it requires much practice and study. A patient who essays to use electricity himself, is unconscious of the fact that there are two kinds of electricity in common use, and several methods of application, each of which is capable of various modifications. Moreover, some constitutions are extremely sensitive to electricity. In nervous exhaustion the effect of galvanism is seen in improvement of sleep, increase of appetite, loss of flatulence and dyspepsia, regulation of the bowels, improvement in the circulatory system, relief of nervousness and mental depression, and of weariness and pain, increased nutrition of the body, and increased aptitude for mental and bodily work. It is, indeed, in such cases as the above that galvanism or electricity is useful. In any organic malady it cannot be beneficial even if not actually the reverse. We have not yet accepted the theory that electricity is the principle of life, even although it may pervade everything. In conclusion, the author observes there is no greater fraud than an electric belt looked at simply from a commercial point of view. A patient bought one the other day, "for which he paid five pounds. The whole thing could have been manufactured at a profit for four or five shillings."

